# Psychometric properties of the Child Health Assessment Questionnaire (CHAQ) applied to children and adolescents with cerebral palsy

**DOI:** 10.1186/1477-7525-6-109

**Published:** 2008-12-04

**Authors:** Nívea MO Morales, Carolina AR Funayama, Viviane O Rangel, Ana Cláudia Frontarolli, Renata RH Araújo, Rogério MC Pinto, Carlos HA Rezende, Carlos HM Silva

**Affiliations:** 1 Associação de Assistência à Criança Deficiente (AACD), Rua da Doméstica, 250, Uberlândia, Minas Gerais, 38413-168, Brazil; 2 School of Medicine, Federal University of Uberlândia (FAMED-UFU), Avenida Para, 1720, Uberlândia, Minas Gerais, 38400-902, Brazil; 3 School of Medicine of Ribeirão Preto, University of São Paulo (FMRP-USP), Av. Bandeirantes, 3900, Ribeirão Preto, São Paulo, 14049-900, Brazil; 4 Rua Martinésia, 303, sala 202, Uberlândia, Minas Gerais, 38400-606, Brazil

## Abstract

**Background:**

Cerebral palsy (CP) patients have motor limitations that can affect functionality and abilities for activities of daily living (ADL). Health related quality of life and health status instruments validated to be applied to these patients do not directly approach the concepts of functionality or ADL. The Child Health Assessment Questionnaire (CHAQ) seems to be a good instrument to approach this dimension, but it was never used for CP patients. The purpose of the study was to verify the psychometric properties of CHAQ applied to children and adolescents with CP.

**Methods:**

Parents or guardians of children and adolescents with CP, aged 5 to 18 years, answered the CHAQ. A healthy group of 314 children and adolescents was recruited during the validation of the CHAQ Brazilian-version. Data quality, reliability and validity were studied. The motor function was evaluated by the Gross Motor Function Measure (GMFM).

**Results:**

Ninety-six parents/guardians answered the questionnaire. The age of the patients ranged from 5 to 17.9 years (average: 9.3). The rate of missing data was low (<9.3%). The floor effect was observed in two domains, being higher only in the visual analogue scales (≤ 35.5%). The ceiling effect was significant in all domains and particularly high in patients with quadriplegia (81.8 to 90.9%) and extrapyramidal (45.4 to 91.0%). The Cronbach alpha coefficient ranged from 0.85 to 0.95. The validity was appropriate: for the discriminant validity the correlation of the *disability index *with the visual analogue scales was not significant; for the convergent validity CHAQ *disability index *had a strong correlation with the GMFM (0.77); for the divergent validity there was no correlation between GMFM and the pain and overall evaluation scales; for the criterion validity GMFM as well as CHAQ detected differences in the scores among the clinical type of CP (p < 0.01); for the construct validity, the patients' *disability index *score (mean:2.16; SD:0.72) was higher than the healthy group (mean:0.12; SD:0.23)(p < 0.01).

**Conclusion:**

CHAQ reliability and validity were adequate to this population. However, further studies are necessary to verify the influence of the ceiling effect on the responsiveness of the instrument.

## Background

Children and adolescents with cerebral palsy (CP) have permanent and non-progressive development disorders. In spite of medical treatment and rehabilitation, several motor limitations can affect functionality and abilities for activities of daily living (ADL) [1].

The need to know the effects of the disease on health conditions and well-being through the eyes of the individual or his/her caretaker has motivated countless efforts to develop more useful instruments to evaluate the impact experienced by patient and their families. These instruments must have appropriate psychometric properties so as to guarantee reliability, validity and sensitivity to changes, and should be easy to apply and to interpret [2,3].

In the past decade health status and health related quality of life (HRQOL) instruments have been developed. Some generic HRQOL questionnaires have already been used in CP patients and have confirmed physical and psychosocial impairment [4-9]. However, few specific instruments (that measure health status or HRQOL) are available for this population and they do not directly approach the concepts related to functionality or ADL [10-15]. Thus, evaluations of these concepts are greatly needed [16].

The Childhood Health Assessment Questionnaire (CHAQ) is a specific instrument that evaluates functional capacity and independence in ADL. CHAQ was constructed to evaluate children and adolescents with juvenile idiopathic arthritis [17], but this instrument has already been applied to patients with current motor limitations due to other chronic diseases like juvenile spondyloarthritis, spina bifida, articular hypermobility, juvenile dermatomyositis, and lupus erythematosus [18-23]. This instrument is easy to apply and interpret and it contains useful concepts for the evaluation of patients with physical limitations like those with CP. The objective of the present study was to verify the psychometric properties of CHAQ as an instrument for the evaluation of children and adolescents with CP.

## Methods

### Participants

Parents or legal guardians of children and adolescents diagnosed with CP aged 5 to 18 years were invited to participate in this cross-sectional study. The study was carried out from December 2003 to April 2004 in a rehabilitation center in the city of Uberlândia, Brazil (Associação de Assistência à Criança Deficiente – AACD). Approval was obtained from the Research Ethics Committee of the center and written consent was obtained from the patients or guardians. A control group representing the healthy population, recruited on the occasion of the validation of the Brazilian version of CHAQ, was also used [17].

Social and demographic data were obtained from the parent/guardian and from the medical files. All patients were submitted to neurological evaluation and classified according to type of clinical manifestation and motor function. Based on the clinical manifestation the patients were distributed into: spastic, extrapyramidal and ataxic. The spastic type was classified as hemiplegia, diplegia and quadriplegia according to motor involvement [24]. The motor function was evaluated according to the Gross Motor Function Classification System (GMFCS) and the patients were grouped into five levels [25]. Epilepsy was diagnosed based on parent report and confirmed by the medical record.

The parents/guardians answered the self-administered CHAQ and were encouraged to fill out the blank items.

The Gross Motor Function Measure (GMFM) was applied by a physical therapist for the evaluation of physical function [26].

### Instruments

#### Child Health Assessment Questionnaire (CHAQ)

CHAQ is a specific instrument initially described as a HRQOL evaluation questionnaire to be used in children and adolescents with juvenile idiopathic arthritis, from the perspective of the parent or patient. But the instrument measures the functional capacity and independence in ADL and has already been applied to patients with other disabling conditions. It was translated, culturally adapted and validated for the Portuguese language to be used in Brazilian children and adolescents with juvenile idiopathic arthritis, from the perspective of the parent or legal guardian [17,27,28].

The questionnaire measures functional capacity and independence during the last week of daily life activities. It is made up of eight domains: *dressing, arising, eating, walking, reach, grip, hygiene *and *activities*. For each domain there is a 4 level difficulty scale that is scored from 0 to 3, corresponding to "without any difficulty" (0), "with some difficulty" (1), "with much difficulty" (2), and "unable to do" (3). The option "not applicable" was also added in the original elaboration of CHAQ; therefore some items were not applied to some younger age groups. The higher scores correspond to the highest degree of incapacity. The average of the scores of the domains makes up the disability index, which varies from 0 to 3 points.

CHAQ also presents two visual analogue scales for pain evaluation and overall well-being evaluation. In the present study, in the last question of the questionnaire that corresponds to the scale of overall evaluation, the word "arthritis" was replaced with "cerebral palsy". This was the only adaptation made in order to apply the instrument to this study population.

The original English version of the CHAQ is available elsewhere [28].

#### Gross Motor Function Measure (GMFM)

GMFM is a specific instrument developed for the purpose of quantitatively measuring the changes in gross motor function that occur in patients with CP over time [26]. It consists of 88 items that are grouped into five dimensions of gross motor function: lie down and roll (17 items), sit down (20 items), crawl and kneel (14 items), stand (13 items), walk, run and jump (24 items). The final score of the instrument is obtained by the average of the scores of the five dimensions, varying from 0 to 100. The highest scores indicate the best function.

GMFM was used as a measure of evaluation of physical function that allowed comparisons with CHAQ.

### Psychometric properties and statistical analysis [29]

Descriptive statistical analysis was used for the demographic and clinical characteristics of patients and informants. The characteristics of the participants and non-participants (individuals who were invited to compose the study group but did not consent or whose evaluations were not concluded) were compared by Student's t-test (for age) and the chi-square test.

The proportion of questionnaires that were not completely filled out (missing data) or items that were not applicable were calculated for each domain and scale, with ideal values being considered to be below 20%. The rates of floor and ceiling effects were calculated as the proportion of patients who obtained the lowest and highest possible scores, respectively, of each domain or scale and were considered to be present when they exceeded 10%.

The Shapiro-Wilk test was used to evaluate the normality of the scores obtained with CHAQ and the normal distribution of the data for both the study and control groups.

Internal consistency reliability was verified by the Cronbach alpha coefficient for each domain.

Item internal consistency was assessed and was considered to be satisfactory if the item achieved the minimum correlation of 0.4 with the domains it represented and if the success rate of the scale was higher than 80%.

The proportion of questionnaires with "not applicable" items was calculated in order to study the face validity. The correlation between questionnaires with "not applicable" items and the following variables was verified: age, classification of clinical type and score obtained by GMFM.

Item-discriminant validity was determined to verify if each item correlated more strongly with the concept it was hypothesized to represent than with different concepts. It was considered satisfactory if the success rate of the scale was higher than 80%.

Discriminant validity is a test of the extent to which one measure is not associated with other measures that are hypothesized as not associated. It was tested by the correlation between domains and disability index (that measures specific aspects of the functional capacity and ADL activities) and the two scales (that measure general aspects of HRQOL and pain). A weak correlation was expected between the domains/disability index and the scale construct.

Convergent validity was determined by the correlation of the CHAQ domains and disability index with the GMFM. A moderate to high correlation was expected. For divergent validity the correlation between the CHAQ scales and the GMFM was tested, and a poor coefficient was expected.

The Pearson correlation coefficient was used for all correlation tests.

Analysis of variance was used to verify the criteria or concurrent validity by comparing GMFM and CHAQ performance according to CP classification. It was expected that both instruments could distinguish could distinguish the motor function limitation of each patient group in the same manner. The Bonferroni test allowed the definition of the differences between the averages of the groups. Patients with ataxia were not included in this analysis due to the small number found in the sample.

Student's t-test was used to determine construct validity by comparing the scores for the patients with those for the control group. The initial hypothesis was that the study population had more functional limitations than the healthy population. The correlation of the patients' GMFCS levels and the CHAQ *disability index *scores was used to confirm the hypothesis that the CHAQ construct has a strong or moderate correlation with the motor function.

## Results

Of the 126 eligible patients, 96 participated in the study. The clinical and demographic characteristics of the patients were similar for participants and non-participants (p > 0.05). No differences were detected between the study and control group according to age (p = 1.15) and gender (p = 0,07). The characteristics of the study group are presented in Table 1.

**Table 1:** Demographic and clinical characteristics of the participants

**Characteristics**	**Participants** **n = 96**
Mean age (SD)	9.3 (3.4)
Male (%)	54 (56.3)
Ethnicity (%)	
- Caucasian	68 (70.9)
- African-Brazilian	28 (29.1)
Classification of CP (%)	
- spastic	81 (84.4)
. *quadriplegia*	*22 (22.9)*
. *diplegia*	*35 (36.5)*
. *hemiplegia*	*24 (25.0)*
- extrapyramidal	11 (11.5)
- ataxic	4 (4.1)
GMFCS	
- level 1	39 (37,5)
- level 2	3 (3.1)
- level 3	20 (20.8)
- level 4	6 (6,25)
- level 5	28 (29.2)
GMFM – mean (SD)	56 (35.1)
Epilepsy (%)	44 (45.8)
Education (%)	
- not receiving education	21 (21.9)
- receiving special education	29 (30.2)
- receiving regular education	46 (47.9)

SD = Standard deviation

The patients were predominantly represented by their mothers (81 0%). The age of the informants ranged from 18 to 61 years (average = 34.8, standard deviation = 8.8). Most of the informants had completed elementary school (52.1%).

### Psychometric properties of CHAQ

#### Data quality

The proportion of missing data was low and varied from 3.1% to 9.3% in the domains and scales (Table 2).

**Table 2:** Data quality: missing data, floor and ceiling effects

**CHAQ**	**Missing data (%)**	**Floor effect (%)**	**Ceiling effect (%)**
Dressing	6.2	3.1	68.8
Arising	3.1	26.0	30.2
Eating	3.1	6.3	45.8
Walking	3.1	13.7	41.1
Hygiene	6.2	3.2	47.9
Reach	7.3	7.4	40.4
Grip	6.2	16.0	40.4
Activities	9.3	2.1	68.1
Evaluation of pain	3.1	35.5	1.1
Evaluation of overall well-being	4.2	26.1	1.1

The floor effect was observed in three domains: *arising *(26.0%), *walking *(13.7%) and *grip *(16%), and was significant in the visual analogue scales (26.1 to 35.5%) (Table 2). Comparison of the scores obtained according to the classification of the clinical type of CP revealed that the floor effect was greater in the hemiparetic group for the *arising *(54.20%) and *walking *(37.5%) domains. In the *grip *domain, the highest proportions occurred in the diparetic and hemiparetic groups (25.7% and 20.8%, respectively). In the visual analogue scales all the groups had high values for the floor effect.

The ceiling effect was detected and was high in all domains (30.2 to 68.8%) and was not present in the visual analogue scales (Table 2). For the quadriplegia group, the rate of the ceiling effect was very high in all domains, ranging from 81.8 to 90.9%. In the extrapyramidal group, the proportion of the ceiling effect was 45.4 to 91.0%, and in the diparetic group it ranged from 14.3 to 65.7%, with higher rates for the *dressing *(62.9%) and *activities *(65.7%) domains. The hemiparetic group showed the lowest ceiling effect rates, which were more significant only for the *dressing *(54.2%) and *activities *(45.8%) domains.

#### Reliability

Reliability was adequate. The Cronbach alpha coefficient ranged from 0.85 to 0.95. The success rate regarding item internal consistency was 100% in all domains (Table 3).

**Table 3:** Reliability: internal consistency reliability and item internal consistency

**Domains**	**Itens (n)**	**Internal consistency reliability^a^**	**Item internal consistency**		
			
			Range of item correlations^**b**^	Success/Total	Success Rate
Dressing	4	0.85	0.44 – 0.95	4/4	100
Arising	2	0.94	0.84 – 0.94	2/2	100
Eating	3	0.85	0.57 – 0.94	3/3	100
Walking	2	0.95	0.86 – 0.89	2/2	100
Hygiene	5	0.95	0.72 – 0.79	5/5	100
Reach	4	0.88	0.50 – 0.76	4/4	100
Grip	5	0.94	0.61 – 0.83	5/5	100
Activities	5	0.90	0.58–0.71	5/5	100

^**a **^Cronbach alpha coefficient

^**b **^Pearson's correlation coefficient

#### Validity

In the determination of face validity, 28.1% of the questionnaires were found to present some "not applicable" items. In 7.3% of the questionnaires there was only a single item considered to be "not applicable", whereas in 9.4% of the questionnaires more than 6 items were "not applicable", i.e., more than 20% of the items were "not applicable". The rate of "not applicable" items according to the domains ranged from 5.2 to 22.9%, and the *activities *domain was the only one that obtained values above 20% (22.9%). There was no correlation between the frequency of "not applicable" items and the variables age, clinical type of CP and score obtained by GMFM (p > 0.05).

The discriminant validity of the item obtained an appropriate success rate in six domains and was below the ideal value for the *dressing *and *activities *domains (Table 4).

**Table 4:** Item discriminant validity

**Domains**	**Itens (n)**	**Range of item correlations^a^**	**Success/Total**	**Success Rate (%)**
Dressing	4	0.34 – 0.95	13/32	40.6
Arising	2	0.35 – 0.94	16/16	100.0
Eating	3	0.32 – 0.94	22/24	91.7
Walking	2	0.41 – 0.89	16/16	100.0
Hygiene	5	0.43 – 0.76	40/40	100.0
Reach	4	0.28 – 0.76	21/24	87.5
Grip	5	0.38 – 0.83	39/40	97.5
Activities	5	0.36 – 0.71	29/40	72.5

^**a**^Pearson's correlation coefficient

For the discriminant validity the correlation of the domains and of the *disability index *with the visual analogue scales was not significant. In general, the domains presented strong to moderate correlations with one another (Table 5).

**Table 5:** Discriminant validity: correlation between CHAQ domains and disability index with the scales

**CHAQ**	**Dressing**	**Arising**	**Eating**	**Walking**	**Hygiene**	**Reach**	**Grip**	**Activities**	**D. Index**	**E. pain**	**E. overall**
**Dressing**	1.00*										
**Arising**	0.29*	1.00*									
**Eating**	0.68*	0.43*	1.00*								
**Walking**	0.43*	0.66*	0.51*	1.00*							
**Hygiene**	0.72*	0.56*	0.66*	0.62*	1.00*						
**Reach**	0.42*	0.47*	0.48*	0.54*	0.55*	1.00*					
**Grip**	0.57*	0.56*	0.65*	0.55*	0.67*	0.44*	1.00*				
**Activities**	0.51*	0.38*	0.46*	0.53*	0.55*	0.33*	0.50*	1.00*			
**D. Index**	0.72*	0.74*	0.78*	0.77*	0.86*	0.69*	0.82*	0.66*	1.00*		
**E. pain**	-0.04	0.05	0.00	0.01	-0.05	0.20	0.08	0.06	0.07	1.00*	
**E. overall**	0.10	0.06	0.09	0.12	0.13	0.07	0.11	0.10	0.17	0.52*	1.00*

*Pearson's correlation coefficient was significant at the 0.01 level

D. Index. = Disability Index; E. pain = Evaluation of pain; E. overall = Evaluation of overall well-being

The convergent validity was satisfactory because GMFM presented a significant correlation with the CHAQ domains and a strong correlation with the *disability index *(0.77). The divergent validity was confirmed because there was no correlation between GMFM and the pain and overall evaluation scales (Table 6).

**Table 6:** Convergent and divergent validity: correlation between CHAQ and GMFM

**CHAQ**	**Correlation with GMFM (r)**
Dressing	-0.43*
Arising	-0.79*
Eating	-0.56*
Walking	-0.72*
Hygiene	-0.65*
Reach	-0.53*
Grip	-0.57*
Activities	-0.41*
Disability Index	-0.77*
Evaluation of pain	-0.14
Evaluation of overall well-being	-0.19

*Pearson's correlation coefficient was significant at the 0.01 level

For the criterion validity it was observed that GMFM as well as CHAQ detected differences in the scores among the groups classified according to the clinical type of CP (p < 0.01), except for the visual analogue scales (p > 0.05). Like the GMFM, the *disability index *and the *arising *domain of CHAQ discriminated the differences among all clinical types of CP analyzed. The *walking *domain also detected differences among the three spastic subtypes of the disease. Patients with quadriplegia presented more physical incapacities as determined by both instruments and in all CHAQ domains (Table 7).

**Table 7:** CHAQ and GMFM mean scores, according to the CP classification

**CHAQ**	**Spastic**			**Extrap** **(n = 11)**	p value*
			
	**Quadri** **(n = 22)**	**Dip** **(n = 35)**	**Hemi** **(n = 24)**		
Dressing	2.91^a^	2.43^ab^	2.33^b^	2.91^a^^b^	0.01
Arising	2.86^a^	1.49^b^	0.62^c^	2.09^d^	0.00
Eating	2.82^a^	2.00^b^	1.83^b^	2.36^ab^	0.00
Walking	2.86^a^	2.26^b^	0.87^c^	2.18^b^	0.00
Hygiene	2.90^a^	2.21^bc^	1.79^b^	2.64^ac^	0.00
Reach	2.90^a^	2.00^b^	1.83^b^	2.36^b^	0.00
Grip	2.90^a^	1.68^b^	1.50^b^	2.36^a^	0.00
Activities	2.90^a^	2.50^ab^	2.25^b^	2.82^a^	0.01
Disability Index	2.90^a^	2.03^b^	1.64^c^	2.47^d^	0.00
Evaluation of pain	0.67^a^	0.30^a^	0.59^a^	0.11^a^	0.09
Evaluation of overall well-being	0.74^a^	0.43^a^	0.48^a^	0.57^a^	0.59

**GMFM**	10.59^a^	63.51^b^	89.50^c^	42.45^d^	0.00

*ANOVA. Mean scores followed by the same letter do not differ from each other by the Bonferroni post hoc test.

Quadri = quadriplegia; Dip = diplegia; Hemi = hemiplegia; Extrap = extrapyramidal

The hypothesis determined in the construct validity that children and adolescents with CP have higher scores, or in other words, more incapacity than the healthy population was confirmed (p < 0.01) in all the CHAQ domains, scales and *disability index *(Table 8).

**Table 8:** CHAQ mean scores for the patient and healthy groups

**CHAQ**	**Mean (SD)**		**Differences among mean scores**	p value*
			
	**Healthy** **(n = 314)**	**Patient** **(n = 96)**		
Dressing	0.34 (0.66)	2.53 (0.79)	2.19	0.00
Arising	0.01(0.13)	1.62 (1.17)	1.61	0.00
Eating	0.16 (0.42)	2.18 (0.92)	2.02	0.00
Walking	0.00 (0.00)	2.03 (1.04)	2.03	0.00
Hygiene	0.08 (0.32)	2.28 (0.82)	2.20	0.00
Reach	0.10 (0.31)	2.15 (0.89)	2.05	0.00
Grip	0.08 (0.35)	1.97 (1.08)	1.89	0.00
Activities	0.20 (0.47)	2.56 (0.73)	2.36	0.00
Disability Index	0.12 (0.23)	2.16 (0.72)	2.04	0.00
Evaluation of pain	0.02 (0.20)	0.42 (0.65)	0.40	0.00
Evaluation of overall well-being	0.01 (0.07)	0.53 (0.62)	0.52	0.00

* Student t test

SD = Standard deviation

A strong correlation of the patients' GMFCS levels and the CHAQ *disability index *scores was obtained (r = 0.73).

## Discussion

The results of the present study demonstrate that the psychometric properties of the Brazilian version of CHAQ were appropriate as a whole for the evaluation of HRQOL in children and adolescents with CP, with possible limitations related to the presence of a significant ceiling effect.

The rate of missing data was low, as also observed for the healthy Brazilian population and for subjects with juvenile idiopathic arthritis [17], indicating good acceptability and effort efforts by the informants in filling out the questionnaires.

The low frequency of the floor effect in the domains suggests that the instrument is able to evaluate and to discriminate patients with smaller motor incapacity. The floor effect was greater for the *arising, walking *and *gripping *domains only for the patients with the hemiparetic form of the disease, and only for the *gripping *domain for the patients with the diparetic form, i.e., this occurred for the tasks executed with less difficulty by these children/adolescents. In the visual analogue scales the floor effect was significant in all the clinical forms of the disease, a fact that may limit the evaluation of patients with less impairment and a lower frequency of pain as perceived by the parent/guardian.

The ceiling effect found in all domains suggests the possibility of the instrument being insensitive to verify differences in HRQOL among the patients with greater motor incapacity. Nevertheless, the instrument was as effective in detecting differences in HRQOL between groups, as GMFM, the instrument used as an external criterion for the evaluation of physical function.

The predominance of the ceiling effect in the quadriplegia and extrapyramidal group was expected since these patients have more motor limitations and the instrument used in the present study covers very specific functional abilities. The great heterogeneity of the population studied hinders the elaboration of an appropriate questionnaire for the whole spectrum of possible motor manifestations in this disease. The evaluation of HRQOL should be complemented with more specific instruments for the patient with greater motor difficulties caused by CP [11,14].

The variability of the scores obtained with the instruments of HRQOL is an indicator of good sensitivity in detecting changes in health conditions. Because this was a cross-sectional study, one of its limitations was the impossibility to test the sensitivity and responsiveness of the instrument. Prospective studies are necessary to evaluate this property and to verify the influence of the floor and ceiling effects on the sensitivity and responsiveness of CHAQ in children and adolescents with CP over time or after interventions. For a future longitudinal study the necessity to include the quadriplegic group should be verified, as CHAQ is an instrument that focuses on daily activities, and we do not expect to have a significant modification with the treatment program in this dimension for this group (we should consider the very high CHAQ scores, in all domains, with many ceiling effects to reinforce this idea). Others instruments with others dimensions could be more useful to evaluate the outcome of the quadriplegic group. But in this cross-sectional study we believe that it was important to evaluate all motor forms of cerebral palsy because it shows us that from the caregiver perspective these patients are very different in the domains measured by this instrument.

In general, CHAQ has been used to evaluate patients with juvenile idiopathic arthritis and musculoskeletal diseases, populations in which the percentage of individuals with lower motor incapacity is high, generating a considerable floor effect and an insignificant ceiling effect [17,19]. Modifications in the options of answers have already been proposed by Lam et al. [19] for the evaluation of patients with musculoskeletal diseases in order to improve the sensitivity of the instrument and its ability to distinguish between patients with milder motor difficulties and the control groups. For the specific population with CP, changes could be made in the questionnaire in order to reduce the ceiling effect and to improve the differentiation of more seriously affected individuals.

In spite of these considerations, the results of the present study demonstrated that the instrument was capable of detecting differences among all the types of CP for the *disability index *and for the *arising *domain. Most of the domains detected more difficulties in the quadriplegia group compared to the diparetic and hemiparetic groups, although they did not differentiate the latter groups from one another, except for the *arising *and *walking *domains. Limitations were observed in the visual analogue scales which are more generic and subjective.

Reliability was found to be appropriate for all domains and the variations found in the correlation coefficient between the items and the domain itself did not suggest redundancy in the questions. The validity was also shown to be generally appropriate for the aspects tested.

In the evaluation of the face validity the instrument was considered appropriate for the study population on the basis of the perception of the informant. The face validity is the extent to which a measure "looks like" what it is intended to measure [29]. In other words, to verify this validity it is necessary to ask the respondent, during completion of the measure, whether the items and scales look reasonable at "face value".

The category of "not applicable" answers was introduced in the original elaboration of CHAQ as an option for younger children, although each domain presents at least one question that can be answered by children under nine years. However, we believe that further information can be obtained when analyzing the proportion of "not applicable" items, because this type of answer suggests inadequacy of the question which is not due only to the influence of the age factor but also to the motor limitation of the patient. Therefore the proportion of questionnaires with "not applicable" items for each domain was analyzed and shown to be useful in the evaluation of face validity in the present study. If the parents/guardians say that the item is "not applicable" we need to think about the value of this question for these patients. The opportunity to have this option in the original version of CHAQ and to use it to access the face validity was very important. It was the first time that this option was used for this purpose in the instrument but future studies should not miss the opportunity offered by the instrument.

For the study population, the presence of "not applicable" questions was expected considering the age range evaluated and the motor limitation of the patient. Although this type of answer was frequent in the study population as a whole, the proportion of questionnaires with more than 20% of "non-applicable" items was low and the value was a little higher only in the *activities *domain. Since the frequency of "not applicable" items was low, when considering the questionnaire as a whole, the correlations of this type of answer with age, clinical type and physical function determined by GMFM were not significant. The values obtained demonstrate that CHAQ is adequate for the evaluation of the functional capacity of children and adolescents with CP as a whole, according to the perception of the parents/guardians.

In the evaluation of the discriminant validity of the items the success rate in the *dressing *and *activities *domains was below the ideal level. Since this is a specific instrument, different from multidimensional questionnaires, it is understood that some items may correlate with more than one domain. For the Brazilian population with juvenile idiopathic arthritis and for healthy controls, the discriminant validity of the items failed in the *dressing*, *walking *and *reaching *domains [17]. These data may suggest the need to review some items and to rearrange them into more homogeneous domains according to the concepts involved, but this does not represent a limitation of the use of the instrument.

From the discriminant validity it was expected that the instrument could discriminate different constructs. Actually, the analysis showed that the visual analogue scales really evaluate concepts that differ from the domains and the *disability index*, with non-significant correlations between them. Moderate and significant correlations among the domains were expected because a specific instrument only involving the physical dimension in the evaluation of functional capacity was used. These concepts were again confirmed when correlating GMFM, the specific instrument for the evaluation of physical function, with the CHAQ domains which corresponded to appropriate convergent validity. The absence of correlation of GMFM with CHAQ scales confirmed the different natures of the measured constructs and demonstrated appropriate divergent validity.

Moreover, GMFM served as an external criterion to verify differences among the clinical types of CP. CHAQ proved to be capable of detecting these differences in all domains, but mainly for the *disability index *and for the *arising *domain. The visual analogue scales were not as useful as the GMFM in the evaluation of the clinical types of CP. This result was expected because GMFM was not considered an external criterion for these scales since they deal with different domains.

The hypothesis raised for construct validity was satisfied, because CHAQ proved to be useful to discriminate the performance of the healthy population and the patients with CP as a whole in all the domains and scales and the *disability index*.

The high but not perfect correlation between *disability index *and GMFCS levels in the present study indicates that CHAQ has a strong correlation with the gross motor function, but it is built to measure others aspects of the physical construct, as hypothesized.

It is essential to examine the measuring properties of the instruments used in the evaluation of health status or HRQOL for the interpretation of the results and for the best applicability of these instruments in clinical practice.

The present study should be interpreted by considering possible inherent methodological limitations. Although CHAQ can be answered by the patient, in this study only the information provided by the parent/guardian was considered. Most of the studies of this nature generally resort to a relative to obtain information. Few studies have obtained the perception of the patient with cerebral palsy and they did not involve representatives of the total population suffering from this disease [15,30,31]. When working with children with developmental disorders, frequently not only physical but various other levels of communication delay, cognitive deficit, learning disability make the presence of a representative essential [2,32]. Due to these limitations, the presence of a representative of the child or of the patients with developmental disorders has the advantage of providing further information about the health conditions and well-being of the patients in addition to the perspective of the health team, even if that implies a potential risk of increasing subjectivity.

Future studies should be conducted to determine the possibility of applying CHAQ directly to the patients with CP, although patients with cognitive limitations should be excluded. The psychometric properties should also be analyzed again for each population group studied.

Others instruments more frequently used in patients with CP to measure the child's performance by parent report like the Pediatric Evaluation of Disability Inventory (PEDI) and the Functional Independence Measure for children (WeeFIM) include a self-care scale [5,16] and they also show a high correlation with GMFM and GMFCS. The Pediatric Quality of Life Inventory (PedsQOL) – Cerebral Palsy Module, a HRQOL specific instrument, has adequate reliability and validity but only includes few questions about ADL [15]. So, these instruments do not provide information about abilities for activities of daily living they are only available in English. CHAQ is a more specific instrument and it is available in at least 32 countries [28]. It would be useful to apply it in association with a generic HRQOL instrument.

## Conclusion

CHAQ reliability and validity were adequate to evaluate children and adolescents with cerebral palsy. However, further studies are necessary to verify the influence of the ceiling effect on the responsiveness of the instrument, mainly in the evaluation of patients with quadriplegia.

## Abbreviations

ADL: activities of daily living; CP: Cerebral palsy; GMFCS: Gross Motor Function Classification System; GMFM: Gross Motor Function Measure; HRQOL: Health related quality of life.

## Competing interests

The authors declare that they have no competing interests.

## Authors' contributions

NMOM conceived the idea, participated in data collection, analyzed and assisted in interpretation of the results and formatted the manuscript. CHMS and CARF conceived the idea, assisted in interpretation of the results and commented on drafts. ACF and RRHA were involved in data collection and assisted in interpretation of the results. VOR and CHAR assisted in analyzing and interpreting the results. RMCP analyzed and assisted in interpreting the data. All authors read and approved the final manuscript.

## References

[B1] Beckung E, Hagberg G (2002). Neuroimpairments, activity limitations, and participation restrictions in children with cerebral palsy. Dev Med Child Neurol.

[B2] Bjornson KF, McLaughlin JF (2001). The measurement of health-related quality of life (HRQL) in children with cerebral palsy. Eur J Neurol.

[B3] Guyatt GH, Naylor D, Juniper E, Heyland DK, Jaeschke R, Cook D (1997). How to use articles about health-related quality of life: evidence-based medicine working group. JAMA.

[B4] Liptak GS, O'Donnell M, Conaway M, Chumlea WC, Wolrey G, Henderson RC, Fung E, Stallings VA, Samson-Fang L, Calvert R, Rosenbaum P, Stevenson RD (2001). Health status of children with moderate to severe cerebral palsy. Dev Med Child Neurol.

[B5] McCarthy ML, Silberstein CE, Atkins EA, Harryman SE, Sponseller PD, Hadley-Miller NA (2002). Comparing reliability and validity of pediatric instruments for measuring health and well-being of children with spastic cerebral palsy. Dev Med Child Neurol.

[B6] Samson-Fang L, Lung E, Stallings VA, Conaway M, Worley G, Rosenbaum P, Calvert R, O'Donnell M, Henderson RC, Chumlea WC, Liptak GS, Stevenson RD (2002). Relationship of nutritional status to health and societal participation in children with cerebral palsy. J Pediatr.

[B7] Wake M, Salmon L, Reddihough D (2003). Health status of Australian children with mild to severe cerebral palsy: cross-sectional survey using the child health questionnaire. Dev Med Child Neurol.

[B8] Morales NMO, Silva CHM, Frontarolli AC, Araújo RRH, Rangel VO, Pinto RMC, Morales RR, Gomes DC (2007). Psychometric properties of the initial Brazilian version of the CHQ-PF50 applied to the caregivers of children and adolescents with cerebral palsy. Qual Life Res.

[B9] Vargus-Adams J (2005). Health-related quality of life in childhood cerebral palsy. Arch Phys Med Rehabil.

[B10] Mackie PCO, Jessen EC, Jarvis SN (1998). The lifestyle assessment questionnaire: an instrument to measure the impact of disability on the lives of children with cerebral palsy and their families. Child Care Health Dev.

[B11] Schneider JW, Guruchari LM, Gutierrez AL, Gaebler-Spira DJ (2001). Health-related quality of life and functional outcome measures for children with cerebral palsy. Dev Med Child Neurol.

[B12] Hammal D, Jarvis SN, Colver AF (2004). Participation of children with cerebral palsy is influenced by where they live. Dev Med Child Neurol.

[B13] Tsirikos AI, Chang WN, Dabney KW, Miller F (2004). Comparison of parents' and caregivers' satisfaction after spinal fusion in children with cerebral palsy. J Pediatr Orthop.

[B14] McCoy RN, Blasco PA, Russman BS, O'Malley JP (2006). Validation of a care and comfort hypertonicity questionnaire. Dev Med Child Neurol.

[B15] Varni JW, Burwinkle TM, Berrin SJ, Sherman SA, Artavia K, Malcarne VL, Chambers HG (2006). The PedsQL in pediatric cerebral palsy: reliability, validity, and sensitivity of the Generic Core Scales and Cerebral Palsy Module. Dev Med Child Neurol.

[B16] Meester-Delver A, Beelen A, Hennekam R, Hadders-Algra M, Nollet F (2006). Predicting additional care in young children with neurodevelopmental disability: a systematic literature review. Dev Med Child Neurol.

[B17] Machado CSM, Ruperto N, Silva CHM, Ferriani VPL, Roscoe I, Campos LMA, Oliveira SKF, Kiss MHB, Bica BERG, Sztajnbok F, Len CA, Melo-Gomes JA (2001). The Brazilian version of the childhood health assessment questionnaire (CHAQ) and the child health questionnaire (CHQ). Clin Exp Rheumatol.

[B18] Takken T, Elst E, Spermon N, Helders PJ, Prakken AB, Net J van der (2003). The physiological and physical determinants of functional ability measures in children with juvenile dermatomyositis. Rheumatology (Oxford).

[B19] Lam C, Young N, Marwaha J, McLimont M, Feldman BM (2004). Revised versions of the Childhood Health Assessment Questionnaire (CHAQ) are more sensitive and suffer less from a ceiling effect. Arthritis Rheum.

[B20] Ruperto N, Malattia C, Bartoli M, Trail L, Pistorio A, Martini A, Ravelli A (2004). Functional ability and physical and psychosocial well-being of hypermobile schoolchildren. Clin Exp Rheumatol.

[B21] Moorthy LN, Harrison MJ, Peterson M, Onel KB, Lehman TJ (2005). Relationship of quality of life and physical function measures with disease activity in children with systemic lupus erythematosus. Lupus.

[B22] Selvaag AM, Flato B, Lien G, Sorskaar D, Vinje O, Forre O (2005). Early disease course and predictors of disability in juvenile rheumatoid arthritis and juvenile spondyloarthropathy: a 3 year prospective study. J Rheumatol.

[B23] Brunner HI, Maker D, Grundland B, Young NL, Blanchette V, Stain AM, Feldman BM (2003). Preference-based measurement of health-related quality of life (HRQL) in children with chronic musculoskeletal disorders (MSKDs). Med Decis Making.

[B24] Hagberg B (1989). Nosology and classification of cerebral palsy. Giornale di Neuropsichiatrica Dell' Eta Evolutiva.

[B25] Palisano R, Rosenbaum P, Walter S, Russell D, Wood E, Galuppi B (1997). Development and reliability of a system to classify gross motor function in children with cerebral palsy. Dev Med Child Neurol.

[B26] Russell DJ, Rosenbaum PL, Cadman DT, Gowland C, Hardy S, Jarvis S (1989). The gross motor function measure: a means to evaluate the effects of physical therapy. Dev Med Child Neurol.

[B27] Len CA, Goldenberg J, Ferraz MB, Hilário MOE, Oliveira LM, Sacchetti S (1994). Crosscultural reliability of the childhood health assessment questionnaire. The Journal of Rheumatology.

[B28] Ruperto N, Ravelli A, Pistorio A, Malattia C, Cavuto S, Gado-West L, Tortorelli A, Landgraf JM, Singh G, Martini A (2001). Cross-cultural adaptation and psychometric evaluation of the childhood health assessment questionnaire (CHAQ) and the child health questionnaire (CHQ) in 32 countries. Review of the general methodology. Clin Exp Rheumatol.

[B29] Health Outcomes methodology symposium (2000). Glossary. Medl Care.

[B30] Hodgkinson I, Anjou MC, Dazord CB, Berard C (2002). Qualité de vie d'une population de 54 enfants infirmes moteurs cérébraux marchants. Éstude transversale. Ann Readapt Med Phys.

[B31] Varni JW, Burwinkle TM, Sherman SA, Hanna K, Berrin SJ, Malcarne VL, Chambers HG (2005). Health-related quality of life of children and adolescents with cerebral palsy: hearing the voice of the children. Dev Med Child Neurol.

[B32] White-Koning M, Arnaud C, Bourdet-Loubère S, Colver A, Grandjean H (2005). Subjective quality of life in children with intellectual impairment – how can it be assessed?. Dev Med Child Neurol.

